# Qualitative Insights on Client and Provider Barriers and Facilitators to Using a Novel Online HIV Pre‐ and Post‐Exposure Delivery Model in Kenya

**DOI:** 10.1002/jia2.70133

**Published:** 2026-06-04

**Authors:** Phelix Okello, Nicholas Thuo, Stephanie D. Roche, Arden L. Saravis, Daniel Mwai, Paulami Naik, Catherine Kiptinness, Tabitha Kareithi, Melissa L. Mugambi, Monisha Sharma, Kenneth Ngure, Katrina F. Ortbald

**Affiliations:** ^1^ Partners in Health Research and Development Center for Clinical Research Kenya Medical Research Institute Nairobi Kenya; ^2^ Public Health Science Division Fred Hutchinson Cancer Center Seattle Washington USA; ^3^ Department of Global Health University of Washington Seattle Washington USA; ^4^ School of Public Health Jomo Kenyatta University of Agriculture and Technology Nairobi Kenya

**Keywords:** barriers and facilitators, HIV, Kenya, online PrEP and PEP delivery, post‐exposure prophylaxis, pre‐exposure prophylaxis

## Abstract

**Introduction:**

Online delivery of HIV pre‐ and post‐exposure prophylaxis (PrEP and PEP) could address persistent access barriers, yet implementation across Africa remains limited. The ePrEP Kenya Pilot (NCT05377138) integrated PrEP and PEP services into an existing e‐pharmacy platform and identified client‐ and provider‐level barriers and facilitators to use.

**Methods:**

In the pilot, clinicians screened adults (age 18+) in Nairobi and Mombasa Counties for PrEP and PEP eligibility via telehealth; pharmaceutical technologists courier‐delivered HIV testing services (including self‐testing) and dispensed PrEP or PEP to eligible clients who paid 150–250 KES (∼$1–2 USD) for HIV testing, ≤149 KES (∼$1 USD) for courier delivery and nothing for telehealth consultation or PrEP/PEP drugs. We conducted monthly check‐in calls with providers and, near study endline, in‐depth interviews (IDIs) with purposively sampled clients and all providers. We analysed verbatim call transcripts and IDIs inductively, then mapped identified barriers and facilitators to the Consolidated Framework for Implementation Research (CFIR).

**Results:**

From February to November 2023, we conducted 10 check‐in calls and interviewed 30 clients (10 PEP, 10 PrEP with 1+ refill, 10 PrEP with no refills) and 10 providers (4 clinicians, 6 pharm techs). Clients had a median age of 27 years (IQR 25–30) and providers 28 years (IQR 27–31); 53% (16/30) of clients and 30% (3/10) of providers were female. In the Outer Setting CFIR domain, providers identified motorcycle manoeuvrability as a delivery facilitator but noted that traffic, poor road infrastructure, bad weather and personal safety concerns posed challenges. In the Inner Setting domain, providers identified information‐sharing practices and collegiality as facilitators. In the Individuals domain, clients’ capability, opportunity and motivation to use online PrEP/PEP services was reportedly facilitated by app‐guided HIV self‐testing, broad delivery zones and enhanced privacy, but hindered by low awareness of these services, limited access to internet‐enabled devices, data security concerns and uncertainties around couriers’ pharmacy credentials. Recommendations included reducing client costs, expanding delivery coverage and hours, and offering alternative delivery options (e.g. medication pick‐up lockers).

**Conclusions:**

Online PrEP and PEP delivery is a promising differentiated service model, especially if partially subsidized by third‐party payers. Implementation success will require model adaptations that address logistical, infrastructural and awareness barriers.

**Abbreviations**
HIV: human immunodeficiency virus
HIVST: HIV self‐testing
IQR: interquartile range
KES: Kenyan Shilling
PEP: post‐exposure prophylaxis
Pharm techs: pharmaceutical technologists
PrEP: pre‐exposure prophylaxis
USD: United States Dollar

## 1 Introduction

Private online e‐commerce platforms are expanding across Africa [1, 2] and could be leveraged to deliver HIV services, potentially mitigating client‐level access barriers to traditional clinic‐based HIV services [3, 4]. In many African countries, including Kenya, HIV incidence remains high [5, 6] despite the availability of free, highly effective HIV pre‐ and post‐exposure prophylaxis (PrEP and PEP) in public clinics [7, 8]. Barriers to accessing clinic‐based PrEP/PEP include HIV stigma, transportation costs and lost wages associated with long wait times [9, 10]. Additionally, PEP candidates are sometimes denied service if their exposure is not occupational or linked to sexual assault.

During the COVID‐19 pandemic, online e‐commerce platforms expanded rapidly across Africa [11], offering clients direct delivery of beauty products, electronics, household items, over‐the‐counter medications and prescription drugs. Building on a public−private partnership model we developed for oral PrEP and PEP delivery at brick‐and‐mortar pharmacies in Kenya [12], we adapted and piloted this model for online PrEP and PEP delivery in collaboration with MYDAWA, a private online e‐commerce platform and Kenya's first licensed online pharmacy [13]. The pilot found that online delivery reached PrEP/PEP‐eligible individuals not engaged in traditional facility‐based services (e.g. men, unmarried individuals), that PEP was in high demand (with five‐fold greater uptake than PrEP), and that online PrEP/PEP delivery was acceptable and satisfactory to clients [14].

While this online delivery model has the potential to improve PrEP/PEP access during an era of constrained resources for HIV programming, insights on model implementation remain limited. To address this gap, we qualitatively explored client and provider perspectives on barriers and facilitators to using online PrEP/PEP delivery and identified implementation strategies that could address model weak points and optimize model scale‐up.

## 2 Methods

### 2.1 Study Design

We conducted in‐depth interviews (IDIs) with clients and providers who participated in the ePrEP Kenya Pilot (ClinicalTrials.gov: NCT05377138)—a single‐arm, prospective study implemented for 15 months (October 2022−December 2023) in Nairobi and Mombasa Counties. Details on the online PrEP and PEP delivery model, pilot design and primary outcomes are reported elsewhere [14, 15].

### 2.2 Online PrEP/PEP Delivery

Briefly, the online PrEP and PEP delivery model was supported with telemedicine, HIV self‐testing (HIVST) and courier delivery. Clients were recruited using targeted advertisements on MYDAWA's website, search engines (e.g. Google) and social media (e.g. Instagram). Clients interested in online PrEP/PEP created a MYDAWA account and scheduled a telehealth visit with a remote clinician. Telehealth visits and courier delivery were available every day from 8 AM to 10 PM, with orders placed before 7 PM delivered the same day. During telehealth visits, remote clinicians screened clients for PrEP and PEP eligibility and safety using a prescribing checklist; clients were additionally screened for symptoms of sexually transmitted infections and sexual violence and referred to appropriate services when necessary.

Clients who met the online PrEP/PEP checklist criteria were courier‐delivered an HIV test kit (a self‐test or a rapid diagnostic test) by a pharmaceutical technologist (“pharm tech”) who conducted the rapid test or assisted clients with HIVST, as needed. The e‐commerce platform incorporated a guided HIVST feature that provided clients with step‐by‐step testing instructions and prompted them to upload a photo of their result. Images were analysed by a computer vision algorithm, which delivered real‐time image quality feedback to clients and an interpretation of the test result to remote clinicians via a clinician‐facing portal. If the remote clinician deemed the result negative, they then authorized the pharm tech to dispense PrEP or PEP. Each month, a trained research assistant (RA) facilitated brief check‐in calls with providers about study implementation to ensure compliance with research procedures and address logistical challenges encountered.

The pilot aimed to generate evidence to inform Kenya's Private Sector Engagement Framework for Delivery of HIV Services, which was under development at the time [15]. For this pilot, MYDAWA received free PrEP/PEP drugs from government stock and staff (four clinicians and six pharm techs, paid for by the study) to conduct PrEP/PEP delivery. Clients did not pay anything for drugs but paid 250 KES (∼$2 US dollars [USD]) for an HIV self‐test or 150 KES (∼$1 USD) for a rapid diagnostic test and ≤149 KES (∼$1 USD) for courier delivery.

### 2.3 Sampling and Recruitment

Per the pilot's eligibility criteria, clients were aged 18+ years and eligible to initiate PrEP or PEP via the online model as per the prescribing checklist criteria; providers were aged 18+ years and either licensed clinical officers or pharm techs. Five months into implementation, we used study records to identify and purposively sample 30 clients who: initiated PEP (*n* = 10); initiated PrEP but never refilled (*n* = 10); and initiated PrEP and refilled at least once (*n* = 10). One year into implementation, we invited all study remote clinicians (*n* = 4) and pharm techs (*n* = 6) to participate in IDIs. Prior to receiving IDI invitations, these same providers had participated in monthly check‐in calls (*n* = 10 total) during implementation.

### 2.4 Data Collection

For IDIs, we developed two semi‐structured guides, informed by the updated Consolidated Framework for Implementation Research (CFIR 2.0)—a meta‐theoretical framework of constructs hypothesized to influence implementation success in real‐world settings, organized into five domains: Innovation, Outer Setting, Inner Setting, Individuals and Implementation Process [16, 17, 18]. In the CFIR 2.0, the Capability, Opportunity, Motivation‐Behaviour (COM‐B) model—a behaviour change framework [19]—is incorporated within the Individuals domain. Since providers were paid study staff who could not change the delivery model or implementation, we focused the provider IDI guide on aspects of the Outer and Inner Setting domains (e.g. workflow) that could have helped or hindered their ability to deliver online PrEP/PEP, along with aspects of the delivery model that they felt made it easier or harder for clients to engage with online PrEP/PEP. We focused the client guide on the Individuals domain, soliciting information about factors that influenced their online PrEP/PEP use.

Interviews were led by Kenyan social scientists (authors PO, NBT, ASL and DMT) experienced in qualitative research and conducted virtually or in‐person in Swahili or English. IDIs were audio recorded, transcribed and translated into English, as needed. Additionally, monthly provider check‐in calls were audio recorded, transcribed verbatim and served as a data source to complement insights from the client and provider interviews.

### 2.5 Data Analysis

Given the lack of prior research on online PrEP/PEP delivery in Africa, we opted to analyse the data using inductive content analysis [20]. First, the same team members who conducted the IDIs immersed themselves in the data through repeated readings of the IDI and call transcripts. Next, two team members (authors PO and NBT) conducted independent, open coding on the same subset of transcripts, taking note of facilitators, barriers and recommendations. These analysts compared their coding and refined the codebook categories and definitions through consensus. Remaining transcripts were equally distributed among the four coders (authors PO, NBT, ASL and DMT), who met on a routine basis to discuss and resolve coding challenges, modifying the codebook as needed. Coding was conducted in Dedoose version 9.0.107 (SocioCultural Research Consultants, LLC; USA).

After coding all transcripts, the analysts reviewed the coded passages and collapsed or expanded categories of barriers and facilitators through consensus. Then, they mapped the final barriers and facilitators to select CFIR 2.0 domains and constructs. Through this mapping, we sought to draw connections between our context‐specific findings and broader implementation theory and to describe the identified barriers and facilitators using standard implementation terminology—important for ease of comparison of findings across studies and settings. We also aimed to highlight the various levels at which the barriers and facilitators occurred to help inform subsequent selection of implementation strategies.

### 2.6 Ethical Approval

This study was approved by the Scientific Ethics Review Unit at the Kenya Medical Research Institute (SERU 4406). During clients’ initial PrEP/PEP telehealth visit, they provided electronic informed consent via email or SMS, which included potential participation in qualitative IDIs. Remote clinicians and pharm techs gave written informed consent to participate in IDIs and have their responses in the monthly meetings audio recorded and used as research data.

## 3 Results

### 3.1 Participants

From February to November 2023, we interviewed 10 providers and 30 clients and conducted 10 provider check‐in calls. Providers had a median age of 28 years (interquartile range [IQR] 27–30) and 6 years (IQR 5–9) of experience in their role; most (70%, 7/10) were male (Table 1). Clients had a median age of 27 years (IQR 25–30); half were female (53%,16/30) and 40% were married (12/30)—which is comparable to those accessing online PrEP/PEP in the larger pilot cohort [15], with slightly more women and married individuals in this sample.

**TABLE 1:** Characteristics of online PrEP/PEP clients and providers who completed IDIs.

	Online PrEP/PEP providers, *n* = 10	Online PrEP/PEP clients, *n* = 30
Age, median [IQR]	28 [27, 31]	27.0 [25, 30]
Sex: Female	3 (30%)	16 (53%)
Married	N/A	12 (40%)
Years of education	N/A	16.0 [13.5, 16.0]
Client type		
*PrEP client, no refill*		10 (33%)
*PrEP client, with refill*		10 (33%)
*PEP client*		10 (33%)
Provider type		
*Clinical officer*	4 (40%)	
*Pharm tech courier*	6 (60%)	
Years of experience, median [IQR]	6 (5, 9)	N/A
Months in ePrEP study at time of interview	12 (5, 14)	N/A

Abbreviations: IDIs, in‐depth interviews; IQR, interquartile range; PEP, post‐exposure prophylaxis; PrEP, pre‐exposure prophylaxis.

### 3.2 Provider Barriers and Facilitators to Online PrEP/PEP Delivery

Provider barriers and facilitators to delivering PrEP and PEP via the online model are outlined in Table 2.

**TABLE 2:** Barriers and facilitators to online PrEP/PEP delivery, identified in the CFIR outer and inner setting domains.

CFIR construct	Barrier (−) or facilitator (+)	Illustrative quote	*Contributing factor(s*) and recommendations
**Domain: Outer setting**			
Local conditions	(+) **Motorcycle manoeuvrability**: allowed courier to move through traffic and reach most clients where located. (−) **Heavy traffic, poor road infrastructure, bad weather and personal safety issues**: deliveries sometimes delayed/rescheduled due to these conditions.	A. *“When you are delivering to rural areas you find that don't have a formal place where you can park your bike… in that case, you are not assured of the safety of your bike and also your safety because …some orders, you will be delivering during odd hours, around 9 pm, 10 pm, or even 11 pm*”—**Pharm tech courier, 28 years**	➢ Pharm tech couriers used motorbikes to deliver services. Recommendation(s): Refine routing systems to consider weather/infrastructure issues that may delay couriers; consider factors like delivery urgency (i.e. PEP). Establish alternative pickup points for clients in unsafe or hard‐to‐access areas.
**Domain: Inner setting**			
Communications	(+) **Providers had agreed‐upon information‐sharing practices**: enabled real‐time communication and problem‐solving.	B. *“We have a WhatsApp group where we share these clients so that they are prioritized with the pharm tech riders, and they know where to deliver”*—**Clinical officer, 28 years**	➢ WhatsApp to discuss delivery issues and problem‐solve together in real time.
Relational connections	(+) **Collegiality among providers**: Clinical officers and pharm tech couriers reported working well together; clearly understood roles in online PrEP/PEP delivery.	C. *“I think what was fundamental for this [delivery model's success] was our teamwork, our ability to come up with solutions….”*—**Clinical officer, 28 years**	Recommendation(s): Facilitate joint training sessions for clinical officers and couriers to build collaborative relationships, enabling better communication and problem‐solving.

Abbreviations: CFIR, Consolidated Framework for Implementation Research; PEP, post‐exposure prophylaxis; PrEP, pre‐exposure prophylaxis.

#### 3.2.1 Outer Setting

Providers reported that using motorcycles for courier deliveries facilitated delivery by keeping delivery times short. Motorcycles were highly mobile, enabling pharm techs to circumvent some traffic and overcome limited space for parking at delivery points (**Quote 2A**). Providers reported that fast delivery times particularly benefited clients with a recent potential HIV exposure who needed PEP within 72 hours. However, providers also noted that heavy traffic and poor road infrastructure—especially in urban areas—occasionally led to delivery delays or the need to reschedule delivery. Other challenges identified included deliveries in areas with known security issues, which posed the risk of product or motorcycle theft, as well as risks to pharm tech couriers’ personal safety, especially when making deliveries at night. Lastly, a few pharm techs noted that the rainy season posed safety challenges, as slippery roads increased the risk of accidents.

#### 3.2.2 Inner Setting

Providers reported that effective communication, strong coordination and good collegiality between remote clinicians and pharm techs facilitated delivery. For example, use of WhatsApp enabled quick, timely and audiovisual communication between team members to address delivery issues, confirm HIV test results and carry out timely PrEP/PEP dispensing (**Quote 2B**). Providers also noted that each cadre clearly understood their role in the model and supported one another in their responsibilities (**Quote 2C**).

### 3.3 Client Barriers and Facilitators to Online PrEP/PEP

Client barriers and facilitators to gaining access to and using the online PrEP/PEP model are outlined in Table 3.

**TABLE 3:** Client barriers and facilitators to online PrEP/PEP access and use, identified in the individual CFIR domain.

CFIR construct	Barrier (−) or facilitator (+)	Illustrative quote	Contributing component(s) and recommendations
**Domain: Individual; *Subdomain: Characteristics***			
Capability	(+) **Guided HIVST feature**: helped clients administer and interpret the HIVST.	D. *“The platform has instructions on uploading results because it shows you step by step … You choose the [brand of] self‐test kit you have used … [indicate] if [the result was] negative, positive, or invalid. You upload [a photo of the] results, then you submit.”*—**Pharm tech courier, 26 years**	➢ This feature facilitated step‐by‐step HIVST use, including guidance on result interpretation.
	(−) **Low awareness of online PrEP/PEP due to limited demand generation**: Advertisement mostly reached existing online clients and those with access to internet and social media.	E. *“You have to be somehow tech‐savvy to be able to use it well.”*—**Male PrEP client**, **25 years**	➢ Model lacked effective off‐line marketing strategies. Recommendation: Spread word of online PrEP/PEP through community organizations; target outreach to schools and workplaces.
	(−) **Online platform requires some level of technological savvy**: Some clients lack the skills/knowledge needed to access/use the platform (e.g. navigate to website; create account; adjust video/audio settings).	F. *“Some people feel like booking the consultation is a bit complex because they have to create an account … [which] involves [providing] a name, email address, age, and password … [that] has to have special characters, capital letters, small letters. So, some people are not able to get it right.”*—**Clinical officer, 30 years**	Recommendation: ➢ Create guides (physical or video) that could walk clients through the online PrEP/PEP delivery steps.
Opportunity	(+) **Broad delivery zones and no travel required**.	G. *“[Clients] appreciate the ease of the service in that you don't have to leave the place of work or to ask maybe for a day off, you don't have to travel from point A to B because this service is being delivered to you.”*—**Pharm tech courier**, **27 years**	➢ Online PrEP/PEP delivered in most parts of the participating counties. Recommendation: Continue to enable broad delivery zones; identify strategies to reach zones not covered.
	(±) **Long operating hours, but not 24/7**: did not suit the needs of certain PEP clients.	H. *“Maybe if someone wants the [PEP] medication past 8:00 PM or very late at night, we can't deliver, and probably they are in their 72 hours [PEP eligibility window].”*—**Clinical officer, 27 years**	➢ Telehealth visits/delivery was available every day, 8 AM−10 PM; clients could schedule visits directly using an online appointment booking tool. Recommendation: Hire more staff to operate on shifts and extend delivery hours, ensuring round‐the‐clock availability for clients with urgent needs.
	(±) **Cost**: subsidized but still unaffordable to some.	I. *“We were having many students from the universities, … but we realize … [some] do not have the money capabilities of reordering [refilling] every month. They need 340 shillings every three months to reorder [refill PrEP], and they were not committed to that.”—* **Clinical officer, 28 years**	➢ Clients paid a fee HIV testing and courier delivery but received the telehealth consult and drugs free of charge.
	(−) **Requires internet‐enabled device and internet connection**: Some clients do not access to such devices and/or internet, others have devices with poor‐quality cameras.	J. *“Most clients might be aware of the online PEP/PrEP delivery platform, but they are unable to access it due to lack of a smartphone. This month there were two clients who requested a phone from a friend.”*—**Monthly meeting, March 2023**	➢ Delivery model required clinical officer to verify HIVST results via a photo. Recommendation: Implement programmes to provide smartphones or subsidized internet access to clients in need.
Motivation	(+) **Enhanced privacy**: did not have to worry about running into people they know.	K. *“You do not have to face many people, and tell them what you want. So, with online [PrEP/PEP delivery], I am dealing with someone I am not seeing so it is private in terms of interaction”*.—**Female PrEP client, 21 years**.	➢ Clients could access online PrEP/PEP discreetly from their home. ➢ One‐on‐one telehealth visit; option to turn off camera. ➢ In‐person interaction limited to pharm tech. ➢ HIVST lets clients learn status on their own. ➢ HIV services and PrEP/PEP drugs delivered to client's choice location. ➢ Items delivered in opaque bags bearing only the e‐pharmacy's logo.
	(+) **Quality time with clinicians**: who were not rushed when assessing clients’ PrEP/PEP eligibility.	L. *“We also exhaust everything, and you also make sure you ask them if they have further questions. And when you go to clinics, I don't think that is the same reception they get there. But via phone call and they understand, I mean we speak the same language, so the uptake is a bit higher.”*—**Clinical officer, 28 years**.	➢ Clinicians and pharm techs well‐versed in PEP/PrEP use and management. ➢ The online health centre provided 15‐min consultation slots for clients, which allowed uninterrupted time to engage comprehensively.
	(+) **Reliable and flexible services**: Most clients found the online PrEP/PEP model dependable and flexible, with deliveries occurring on time, requested delivery changes accommodated, and missed deliveries promptly rescheduled.	M. *“[Clients] have an option of rerouting that order. It's just a phone call away that, [When] I had ordered PEP, I said it [should] be delivered at this location, but now I'm headed home. I want it to be rerouted to home and this is the new address.’ That's all they have to do. I don't think you will meet any other [more] flexible platform than that.”* **—Pharm tech courier, 37‐years**	
	(+) **No HIV stigma**: Unlike public health facilities, which tend to offer PrEP/PEP in HIV clinics.	N. *“And when you go to a public facility, many people… would think that you are taking HIV medication and judge you… I think taking it… online is private and no one will be looking at you and wondering what you are going to take there. I think that is the main driver, privacy.”*—**Female PEP client, 26 years**	➢ The ePrEP programme is embedded within a larger e‐pharmacy offering wide variety of health and beauty products.
	(+) **Wrap‐around services**: Because the larger e‐pharmacy sells a wide variety of products, PrEP/PEP clients could get multiple health needs addressed in a single consultation.	O. *“The [e‐pharmacy's] online platform … is so ideal with privacy, convenience …. There was even somebody who needed that PEP [who] also came with a diagnosis of … UTI [urinary tract infection]. Then in that case, when they ordered the PEP, we can also deliver it with the UTI prophylaxis medication.”*—**Pharm tech courier, 30 years**	
	(−) **Data security concerns**: Some clients felt uneasy about uploading sensitive information (e.g. HIVST results) to the online platform for fear it would be leaked.	P. *“[Some clients] fear maybe when uploading [the HIVST result], you are uploading maybe to the public … [and not just] the MYDAWA app. The only people who can see [the result] are the clinicians and the pharm tech rider who has done [i.e., assisted clients with] the test.”* **—Pharm tech courier, 26 years**	Recommendation: Make the data privacy policy available to clients and ensure providers can adequately explain data security measures.
	(−) **Lack of awareness of pharm tech couriers training**: Some clients doubted couriers’ pharmacy backgrounds, perceiving them to be regular delivery personnel, discouraging some clients from obtaining HIVST assistance.	Q. *“PEP guys [clients seeking PEP] are anxious. They are like, ‘No, you cannot send me a stranger to come and check me and I have not certified them [i.e., verified their credentials].”* **—Clinical officer, 28 years**	➢ Pharm tech couriers wore the same company‐issued uniform as other delivery riders. Recommendation: Share pharm tech couriers’ credentials (e.g. copy of current license) to increase client trust.

Abbreviations: CFIR, Consolidated Framework for Implementation Research; HIVST, HIV self‐testing; PEP, post‐exposure prophylaxis; PrEP, pre‐exposure prophylaxis.

#### 3.3.1 Client Capability

Both clients and providers identified the model's guided HIVST feature—which provided clients who opted for HIVST with detailed self‐test use and interpretation instructions—as facilitating their ability to engage in online PrEP/PEP (**Quote 3D**). However, two major barriers that hindered some clients’ ability to use the online model were low awareness of PrEP and PEP availability via this channel and limited technological literacy. Some clients reported that it was difficult to create an online account (a multi‐step process requiring entering personal details) and navigate the online platform (**Quote 3E**)—an observation echoed by one clinical officer (**Quote 3F**).

#### 3.3.2 Client Opportunity

Clients and providers identified the model's broad delivery zones and long operating hours as facilitating clients’ opportunity to engage with online PrEP/PEP, noting that getting HIV testing services and PrEP/PEP drugs delivered directly to them at a location of their choosing helped them circumvent travel‐ and time‐related barriers (**Quote 3G**). Additional convenience came from the telehealth visits, which were available 7 days a week; clients noted that this broad availability meant clients could access services outside of their work hours and on days when clinics are typically closed. Lastly, some clients reported that the price charged in this model—150−250 KES for HIV testing and ≤149 KES for courier delivery—facilitated their use of online PrEP/PEP because they found it affordable. Factors that reportedly hindered some clients’ opportunity to use the online model included the lack of availability of telehealth visits and courier delivery from 10 PM to 8 AM—which was particularly challenging for clients seeking PEP near the end of their 72‐hour eligibility window (**Quote 3H**)—and cost, which some clients said they struggled to afford (**Quote 3I**). Lastly, both providers and clients reported that, by advertising primarily via the e‐pharmacy's website and social media platforms, the model mostly reached existing e‐pharmacy clients and those with internet access who engage with social media, leaving out other populations, such as those without access to an internet‐enabled device (**Quote 3J**).

#### 3.3.3 Client Motivation

Key factors clients reported that motivated them to obtain PrEP/PEP via the online model were privacy and lack of stigma; quality time with remote clinicians; reliability and flexibility of telehealth visits and deliveries; and ability to get other products and services via the e‐commerce platform. Clients noted that, unlike overcrowded health clinics where privacy is often compromised, online PrEP/PEP delivery allowed them to consult with remote providers from a private location of their choice (**Quote 3K**). They further reported liking that the remote clinicians did not rush them but rather took the time to address all their questions and concerns (**Quote 3L**). Clients reported that deliveries were reliable and that they appreciated being able to adjust delivery times or locations, when needed (**Quote 3 M**). Lastly, clients noted that, since the online pharmacy offers diverse products and services besides just those for HIV, they did not feel HIV stigma when accessing online PrEP/PEP services (**Quote 3N**), and they were able to get multiple health needs addressed in a single interaction with the remote clinician (**Quote 3O**). Two factors that reportedly reduced client motivation, however, were data security concerns and uncertainty around the pharmacy credentials of the couriers delivering HIV testing services and PrEP/PEP drugs. For example, some clients felt uneasy sharing sensitive information (e.g. HIV test results) with the e‐commerce platform, citing concerns about data privacy and potential data leaks (**Quote 3P**). Additionally, some clients, not realizing that the couriers were trained pharm techs, were hesitant to accept their help with the HIV testing process (**Quote 3Q**).

### 3.4 Provider and Client Recommendations for Model Improvement

To increase delivery efficiency, providers recommended refining the courier routing system to avoid bad weather and poor infrastructure; establishing alternative pickup points, such as lockers for clients in hard‐to‐reach areas or areas that posed security risks; and prioritizing clients who are potential PEP candidates given their limited window of eligibility. Lastly, providers recommended a joint refresher training session for remote clinicians and pharm techs together to further promote collaboration, communication and effective problem‐solving.

Clients and providers also proposed strategies to address the informational, logistical and trust‐related barriers they observed. To boost awareness, interviewees from both groups suggested collaborating with community‐based organizations for offline outreach. To support less tech‐savvy clients, providers suggested creating printed or digital guides for pharm tech couriers to use to walk clients through the steps of online PrEP/PEP delivery. To reach clients seeking PEP in a timely manner, providers suggested hiring more staff and working in shifts to extend the hours of telehealth visits and deliveries. To reach lower‐income clients, providers suggested collaborating with telecom companies to subsidize internet access or smartphones. Finally, to address data security concerns, providers suggested discussing the data privacy policy during the initial telehealth visit and sharing documentation of this policy with clients; they also suggested having pharm techs carry their license so that they can present it to clients who are unsure about their credentials and/or competency to support HIV testing.

## 4 Discussion

We qualitatively assessed barriers and facilitators to online PrEP/PEP among Kenyan providers and clients, highlighting numerous multi‐level factors influencing implementation success along with potential ways to improve this novel PrEP/PEP delivery model. Key facilitators included the use of motorcycles and strong provider teamwork; barriers included heavy traffic, poor road infrastructure, bad weather and safety concerns. Online PrEP/PEP access facilitators included app‐guided HIVST, broad delivery zones, extended hours, privacy, stigma‐free interactions and holistic care; barriers included low awareness, limited digital access, data security concerns and uncertainty about couriers’ pharmacy credentials. Recommendations for refining online PrEP/PEP to address delivery barriers included altering routing systems to account for weather and infrastructure issues and establishing alternative pickup points in unsafe or hard‐to‐reach areas. Recommendations on model refinements to address access barriers included hiring more staff and working in shifts so service is available at all hours, sharing couriers’ pharmacy credentials with clients to build trust and implementing programmes to provide smartphones or subsidized internet—potentially in partnership with telecom companies.

These qualitative findings complement the quantitative findings from the ePrEP Kenya Pilot study [14]. In quantitative survey data, PrEP and PEP clients reported high acceptability of the online delivery model, along with high satisfaction with the delivery model and high quality of services received [14]. This qualitative research expands upon our quantitative findings by identifying several possible drivers of these positive ratings, such as the convenience, privacy and quality time with providers that the online PrEP and PEP model reportedly offered. This study also identifies challenges not captured in quantitative client survey data (e.g. affordability issues, data security concerns) and additionally gathers perspectives from providers (none of whom were surveyed during the pilot study). These findings aid in understanding model components that supported providers (e.g. established information sharing practices) and identifying areas where providers could receive additional support (e.g. optimized routing systems).

Findings from this pilot are consistent with other pilot studies in the United States, Brazil, Vietnam, Thailand and South Africa [21, 22, 23, 24] that delivered components of PrEP services remotely. Many of these pilot studies were implemented during the COVID‐19 pandemic to maintain PrEP access during lockdowns [24, 25, 26, 27, 28] and most focused on the use of telehealth and courier delivery to support PrEP continuation [25, 27, 28], not initiation. In these studies, PrEP users also reported high acceptability of online PrEP, primarily due to the convenience and privacy of remote delivery [27, 28, 29, 30]. Similar to our findings, these studies also reported client concerns about data security and distrust in unknown, remote providers [31, 32]. Across various online PrEP delivery models, facilitation of HIV testing remained a key challenge. Many of the US‐based online PrEP delivery models used mail‐in self‐collection kits for HIV testing prior to drug dispensing [33, 34, 35]; the use of HIVST in our model streamlined online PrEP delivery, promoted self‐care and addressed an evidence gap on the use of HIVST to support PrEP initiation [36]. Additionally, our model was unique from other pilots by delivering PEP alongside PrEP, with high PEP uptake suggesting that e‐commerce platforms might be particularly well‐suited to deliver this time‐sensitive service.

The strengths of our study—the first to test PrEP and PEP delivery via an e‐commerce platform in Africa—include conducting IDIs with providers and clients, which provided insights into both the delivery of and access to online PrEP/PEP; using data from monthly provider meetings to complement IDI findings; and structuring findings using an established implementation science framework: CFIR. Limitations included only conducting IDIs at a single time point; limited follow‐up during monthly check‐in calls on how barriers/facilitators mentioned in prior calls progressed over time; only interviewing clients dispensed online PrEP or PEP, preventing identification of barriers that may have prevented clients from engaging with the e‐commerce platform (e.g. mistrust; language barriers); and not analysing data by sex to assess differences in online PrEP/PEP use for male versus female clients.

## 5 Conclusions

International funding for HIV prevention programmes across Africa is in steep decline, requiring the identification of new differentiated service delivery models that leverage existing or emergent healthcare platforms to fill implementation gaps. We found that online PrEP and PEP services were well‐liked among both Kenyan providers and clients, with key model facilitators including flexible telehealth visits, timely service delivery and guided HIVST. However, challenges to model scale‐up in Kenya and other similar settings remain, including infrastructure barriers to delivery, limited digital access among potential clients and high costs for some clients. Our findings provide insights for other settings interested in the development, implementation and scale‐up of PrEP and PEP via e‐commerce platforms.

## Author Contributions

KFO, MS, MLM and KN designed the ePrEP Kenya pilot study and obtained funding. MLM, NT, PN, PO, ALS and KN designed the IDI guides. PO, NT, ALS and DMT analysed the data. SDR, KO and KN helped interpret results. PO, NT and KFO wrote the first draft of this manuscript. All authors edited the draft, provided insights and approved the final manuscript for publication.

## Funding

This work was supported by the Bill and Melinda Gates Foundation (INV‐037646). KFO and MS both received additional funding from the National Institute of Mental Health (R00 MH121166; K01 MH115789).

## Conflicts of Interest

The authors declare no conflicts of interest.

## Data Availability

De‐identified individual participant data are available under appropriate data‐sharing agreements to researchers with a methodologically sound proposal.
